# UNIVERSITY KENAN DISTINGUISHED PROFESSOR AND CO-DIRECTOR, PROGRAM ON AGING, DISABILITY, AND LONG-TERM CARE

**DOI:** 10.1093/geroni/igad104.1494

**Published:** 2023-12-21

**Authors:** Sheryl Zimmerman

**Affiliations:** University of North Carolina at Chapel Hill, Chapel Hill, North Carolina, United States

## Abstract

The concept of person-centeredness -- simply stated, knowing the person in a holistic perspective and meeting the individual’s needs and preferences -- has become so ubiquitous in health and supportive care that it borders on being a cliché. More so, it is often not reflected in national surveys and databases; considered in relation to the capacity of varying systems of care; forward-thinking in terms of emerging realities; or dissected in reference to some of the nuanced and implicit issues responsible for bringing it to the forefront of national attention. This Kleemeier Award lecture will reconstruct the concept of person-centeredness based on literature review, questionnaires, interviews, forums, and think tank meetings with diverse experts. It is supported by National Institute of Aging through a grant to the Alzheimer’s Association’s LINC-AD.

